# A black-and-red stick insect from the Philippines – observations on the external anatomy and natural history of a new species of *Orthomeria*

**DOI:** 10.3897/zookeys.559.6281

**Published:** 2016-02-03

**Authors:** Davide Vallotto, Joachim Bresseel, Thierry Heitzmann, Marco Gottardo

**Affiliations:** 1 LCM - Laboratorio di Caratterizzazione Materiali, Department of Philosophy and Cultural Heritage, Ca’ Foscari University, Malcanton Marcorà Dorsoduro 3484d, 30123 Venice, Italy; 2 Royal Belgian Institute of Natural Sciences, D.O. Phylogeny and Taxonomy, Entomology, Vautier Street 29, B-1000 Brussels, Belgium; 3 P.O. Box 2632, Manila CPO, Philippines; 4 Department of Life Sciences, University of Siena, Via Aldo Moro 2, 53100 Siena, Italy

**Keywords:** Insecta, Phasmatodea, Aschiphasmatidae, stick insects, new species, taxonomy, morphology

## Abstract

A new stick insect of the genus *Orthomeria* Kirby, 1904 (Phasmatodea, Aschiphasmatidae) is described from the Philippines. Orthomeria (Orthomeria) kangi **sp. n.** is readily distinguished from all other congeners by the distinctive blood red colouration of the costal region of the hind wings. Major features of the external morphology of adults, eggs, and first-instar nymphs are illustrated. Locomotory attachment pads are of the smooth type with irregular microgrooves on the contact surface. An unusual condition of male terminalia is the absence of tergal thorn pads on segment 10. The male clasping organs are represented by an elongated vomer terminating in a prominent spine, and by incurved cerci featuring a bilobed apex equipped with a sharp blade-like ridge. Intraspecific variation in body colouration and hind wing length occurs in females. The new species lives at 400-650 m elevation in the surroundings of the Sablang and Tuba regions, in the Benguet Province of Luzon island. Host plants include *Ficus* spp. (Moraceae), and *Pipturus* spp. and *Leucosyke* spp. (Urticaceae). Observations on the mating and defensive behaviour are presented. Orthomeria (Orthomeria) catadromus (Westwood, 1859) is recognised as a junior synonym of Orthomeria (Orthomeria) pandora (Westwood, 1859), **syn. n.** A lectotype is designated for both species. Finally, an updated identification key to the species of the subgenus *Orthomeria* is provided.

## Introduction

The stick insect genus *Orthomeria* Kirby, 1904 belongs to the South-east Asian family Aschiphasmatidae (Bragg 2001), and includes ten species divided into two subgenera (Bragg 2006). The nominal subgenus *Orthomeria*, characterised by a relatively thickened body with dark brown or black basic colouration, comprises seven species found in Borneo, Seram, Sulawesi, Sumatra, and the Philippines (Bragg 2001). The subgenus *Parorthomeria*, characterised by a slim green body and setose eggs, contains the remaining three species that are endemic to Borneo (Bragg 2006).

As part of our research on the Philippine stick and leaf insect fauna (Gottardo 2007; Gottardo 2008; Hennemann et al. 2009; Bresseel 2012), a field expedition was carried out to the Benguet province of Luzon island during which several specimens of *Orthomeria* were found. After a careful examination of the general anatomy, the specimens were assumed to represent a new species of the subgenus *Orthomeria* showing an original set of morphological traits, including a distinctive red colouration on the wings which was not previously reported within the genus (see Bragg 2001).

The aim of this study is to provide a formal description of the new species. Some features of the external anatomy such as attachment devices and male terminalia are characterised in detail for the first time in *Orthomeria*. The morphological data are integrated with observations on the habitat and various life traits. We also provide an updated identification key to the species of the subgenus *Orthomeria* adapted from Bragg (2001).

## Material and methods

*Orthomeria* specimens were collected at night by searching the vegetation along road sides. Specimens were euthanized in glass jars with fumes of ethyl acetate, and subsequently preserved dried and pinned. Some adult females were kept alive to obtain eggs. Linear body dimensions were taken with digital calipers (to the nearest 0.1 mm). The description of chromatic characters was based on live specimens. Observations on the external morphology were carried out with a Zeiss Stemi DV4 stereo light microscope. Photomicrographs were taken with a Nikon D200 SLR digital camera equipped with Nikon Micro-Nikkor AI-s 105 mm f/2.8 lens or with Nikon 24 mm f/2.8 AI-s lens. For scanning electron microscopy (SEM) observations, samples were dehydrated through a graded ethanol series and dried with CO_2_ at the critical point (Balzers CPD 030). Dried samples were mounted on aluminium stubs, sputter coated with gold (Balzers MED 010), and observed with a Philips XL20 scanning electron microscope operating at an accelerating voltage of 10 kV.

The wing venation nomenclature follows Ren (1997). Terminology of eggshell features follows Clark Sellick (1997).

The following acronyms are used to designate the collections: BMNH - The Natural History Museum, London, United Kingdom; MSNG – Museo Civico di Storia Naturale “Giacomo Doria”, Genova, Italy; OC – Private collection Oskar V. Conle, Oberstaufen, Germany; RBINS – Royal Belgian Institute of Natural Sciences, Brussels, Belgium; UMO – University Museum, Hope Entomological Collections, Oxford, United Kingdom; UPLBM – Museum of Natural History, University of the Philippines at Los Banos, Laguna, Philippines.

## Results

### Orthomeria (Orthomeria) kangi sp. n.

Taxon classificationAnimaliaORDOFAMILIA

http://zoobank.org/52E1ED01-9054-4983-BD5A-82B9187EA8B3

#### Holotype.

**Figure 1.**
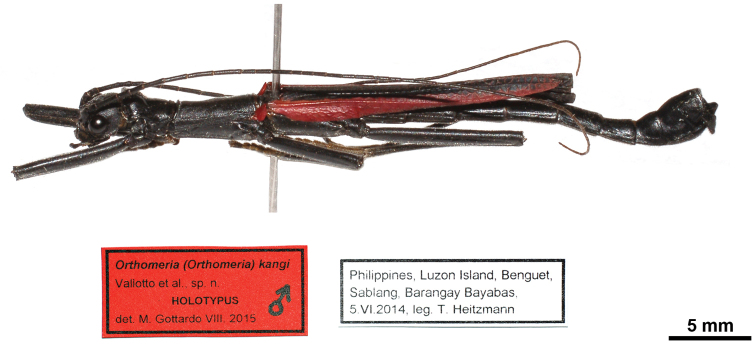
Orthomeria (Orthomeria) kangi sp. n. holotype ♂, habitus, lateral view.

1 ♂, Philippines, Luzon Island, Benguet, Sablang, Barangay Bayabas, 5.VI.2014, leg. T. Heitzmann (MSNG) (Fig. 1).

#### Paratypes.

1 ♂, 2 ♀♀, and eggs (MSNG); 1 ♂, 1 ♀, and eggs (OC); 1 ♂, 1 ♀, and eggs (RBINS); 1 ♂, 1 ♀, and eggs (UPLBM), same data as for holotype.

#### Additional material examined.

3 ♂♂, 3 ♀♀, eggs (reared by D. Vallotto 2015; origin, same locality as for holotype; used for macrophotography); 2 ♂♂ (reared by M. Gottardo 2015; origin, same locality as for holotype; used for SEM).

#### Etymology.

This species is named after Albert Kang (Manila) who discovered the first specimens during a photographic trip down the Benguet province of the Philippines.

#### Diagnosis.

A new species of Orthomeria (Orthomeria) characterised by (1) relatively small body size, ♂♂ ca. 40 mm in length or shorter, ♀♀ less then 50 mm in length; (2) tegmina blood red; (3) costal area of hind wings with blood red markings; (4) tergum 7 of ♀♀ pale brown with a definite black longitudinal line centrally; (5) relatively short hind wings, only slightly projecting over abdominal tergum 5.

#### Description of the male.

A full set of measurements is presented in Table 1.

**Table 1.** Morphometric data for the type specimens of Orthomeria (Orthomeria) kangi sp. n. from Benguet, Philippines.

Parameter	♂ holotype	♂♂ paratypes	♀♀ paratypes
*Measurement (mm)*			
Body length	38.8	37.3–40.1	41.8–46.2
Antenna length	38.4	34.3–35.3	38.7–41.0
Head length	3.0	2.8–3.3	3.8–5.5
Pronotum length (PL)	3.6	2.8–3.2	3.9–4.5
Mesonotum length (MOL)	4.9	4.4–4.8	5.7–6.9
Metanotum length (MAL)	2.2	1.9–2.3	2.6–2.8
Median segment length (MSL)	3.7	3.5–4.0	4.1–4.5
Tegmina length	1.3	1.1–1.3	0.9–1.6
Hind wing length	19.0	17.0–18.4	14.9–18.9
Fore femur length	6.9	6.9–7.0	6.8–7.9
Fore tibia length	6.0	6.0–6.1	6.4–7.4
Mid femur length	6.4	5.7–6.0	6.3–6.7
Mid tibia length	5.8	5.4–5.7	6.1–6.7
Hind femur length	9.6	8.6–9.6	8.7–10.1
Hind tibia length	9.4	9.0–9.3	9.4–10.2
Cercus length	1.9	1.9–2.3	1.2–1.4
*Morphometric ratios*			
MOL divided by PL	1.36	1.50–1.57	1.46–1.53
MAL divided by MOL	0.45	0.43–0.48	0.41–0.46
MSL divided by MAL	1.68	1.74–1.84	1.58–1.61

**Figure 2.**
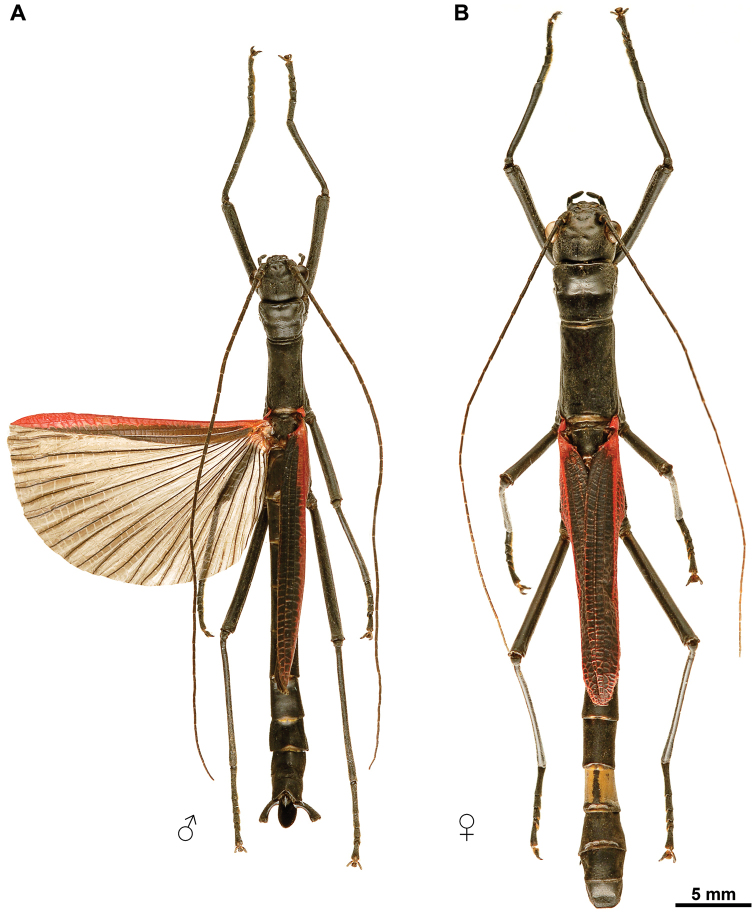
Orthomeria (Orthomeria) kangi sp. n. habitus, dorsal view **A** Adult ♂ **B** Adult ♀.

Colouration: Body, compound eyes, antennae and legs black. Tegmina red. Costal region of hind wings blood red with a longitudinal black narrow stripe on posterior margin. Anal region of hind wings dark brown (Figs 1, 2A).

**Figure 3.**
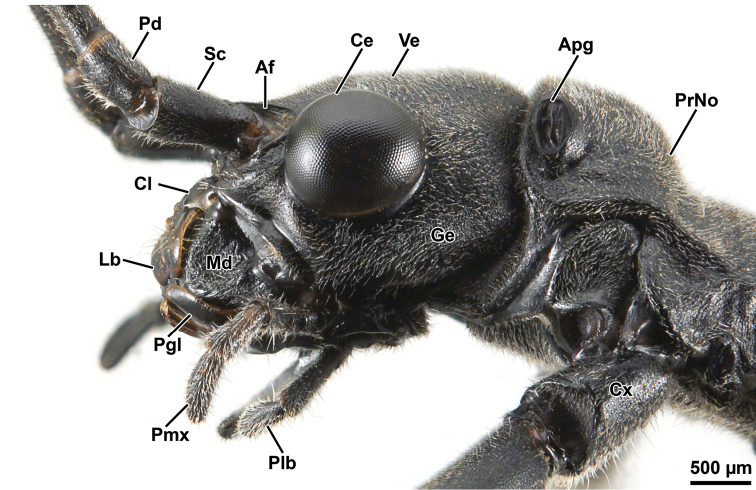
Orthomeria (Orthomeria) kangi sp. n. ♂ head and prothorax, lateral view; Apg, aperture of pronotal gland; Af, antennal field; Ce, compound eye; Cl, clypeus; Cx, coxa; Ge, gena; Lb, labrum; Md, mandible; Pd, pedicellus; Pgl, paraglossa; Plb, labial palpus; Pmx, maxillary palpus; PrNo, pronotum; Sc, scapus.

**Figure 4.**
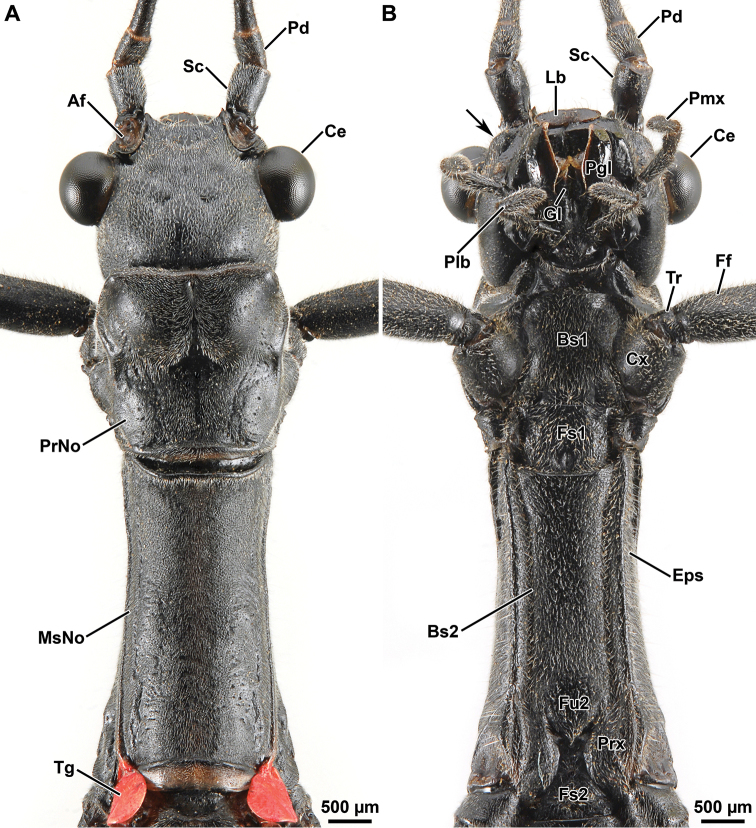
Orthomeria (Orthomeria) kangi sp. n. ♂ head and pro- and mesothorax **A** dorsal view **B** ventra view; Af, antennal field; Bs1-Bs2, pro- and mesothoracic basisterna; Ce, compound eye; Cx, coxa; Eps, episternum; Ff, fore femur; Fs1-Fs2, pro- and mesothoracic furcasternites; Fu2, mesothoracic furca; Gl, glossa; Lb, labrum; Pd, pedicellus; Pgl, paraglossa; Plb, labial palpus; Pmx, maxillary palpus; PrNo, pronotum; Prx, precoxale; Sc, scapus; Tg, tegmina; Tr, trochanter; Arrow, frontal convexity.

Head: In lateral view (Fig. 3), head capsule prognathous, almost as long as the pronotum. Vertex relatively flat. Compound eye large and circular, distinctly protruding hemispherically. Gena narrower than the diameter of the compound eye. Paraglossae not distinctly surpassing the anterior margin of the labrum. In dorsal view (Fig. 4A), head capsule slightly wider than long; dorsal surface with four shallow median depression between the compound eyes. Coronal suture barely recognizable. Ocelli lacking. In ventral view (Fig. 4B), frontal convexity ventrad the antennal base present. Labrum notched anteromedially. Median gular sclerite present and small (not shown). Palpomeres of labial and maxillary palps cylindrical. Antenna filiform, slightly shorter than body length (Fig. 2A), consisting of ca. 58 antennomeres; scapus roughtly rectangular; pedicellus cylindrical, shorter than scapus (Fig. 4); first flagellomere twice as long as pedicel. Antennifer absent.

**Figure 5.**
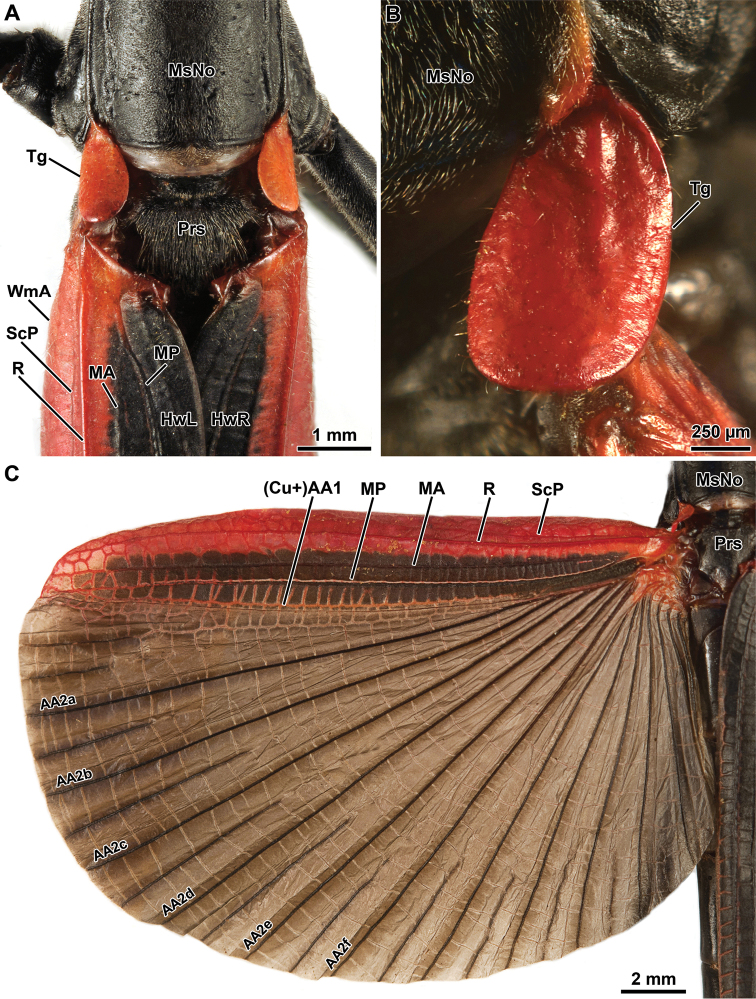
Orthomeria (Orthomeria) kangi sp. n. ♂ **A** Pterothorax, dorsal view **B** Right tegmina, lateral view **C** Left hind wing, dorsal view; AA2a-f, second anterior Analis; (Cu+)AA1, Cubitus + first anterior Analis; HwL, Left hind wing; HwR, right hind wing; MA, anterior Media; MP, posterior Media; MsNo, mesonotum; Prs, prescutum; R, Radius; ScP, posterior Subcosta; Tg, tegmina; WmA, anterior margin of the hind wing.

Thorax: Prothorax longer than head. Pronotum longer than wide (Fig. 4A); anterior half approximately one-third higher than posterior half (Fig. 3); front margin very moderately concave with narrow elliptical glandular field laterally (Fig. 3); hind margin rather straight. Paranota well-developed. Prothoracic coxopleurite subtriangular; prothoracic basisternum with bell-shaped outline (Fig. 4B). Mesothorax very moderately constricted anteriorly, then slightly widening posteriorly. Mesonotum with concave front and hind margins, about 1.70 times length of pronotum. Basisternum flat; precoxale narrow with subtriangular outline; furcasternum with clearly demarcated furca (Fig. 4B). Metathorax only slightly shorter then mesothorax. Metanotum with globose and strongly setose prescutum (Fig. 5A). Metathoracic pleural and sternal regions as in the mesothorax but shorter.

Wings: Tegmina very small, oval, without shoulder pads (Fig. 5A–B). Hind wing slightly extending beyond the fifth abdominal tergum (Figs 1, 2A). Area between anterior wing margin and posterior Subcosta bent laterally; posterior Subcosta weak, not reaching the wing apex; Radius strongly sclerotized and unbranched, parallel to posterior Subcosta; anterior Media and posterior Media simple and straight; Cubitus + first anterior Analis straight; hind wing fan oval (Fig. 5C).

**Figure 6.**
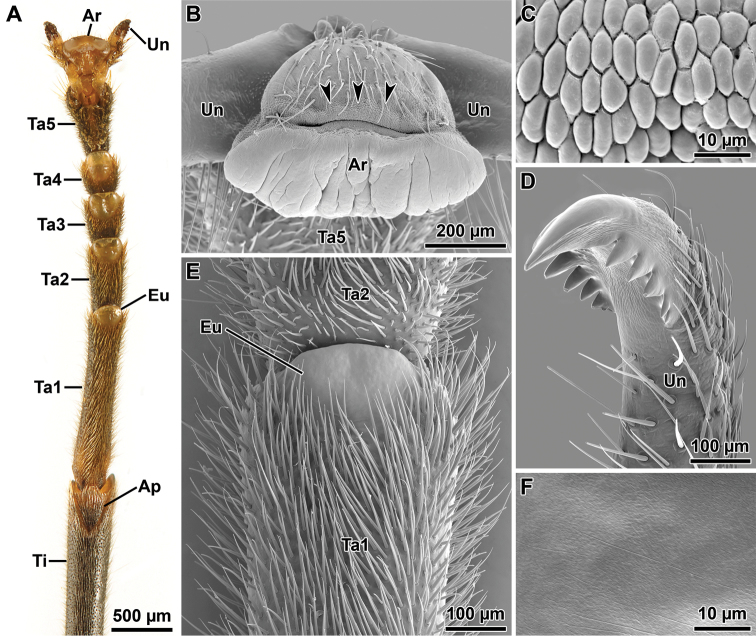
Orthomeria (Orthomeria) kangi sp. n. tarsal and pretarsal attachment devices **A** Fore tarsus, ventral view **B** Arolium, frontal view, arrowheads indicate the outer band **C** Arolium’s micropattern at the outer band level **D** Pretarsal claw, lateral view **E** Tarsal euplantula, ventral view **F** Euplantula’s micropattern; Ap, area apicalis; Ar, arolium; Eu, euplantula; Ta1-Ta5, tarsomeres 1-5; Ti, tibia; Un, peretarsal claws.

Legs: Hind leg distinctly projecting beyond the abdomen (Fig. 2A). Coxa unarmed; trochanters small and fused to femora (Fig. 4B). Femora semicircular in cross section with weakly developed carinae. Fore femur straight basally, unarmed (Fig. 2A). Mid femur with 3 minute spines on the ventro-anterior carina. Hind femur with 4–7 small spines on the ventro-anterior carina and about 3 minute spines on the ventro-posterior carina. Tibiae unarmed and circular in cross section, lacking carinae, with V-shaped area apicalis (Fig. 6A). Tarsus more than two-thirds the length of the corresponding tibia (Fig. 2A). Proximal tarsomere (1) elongated, about as long as combined length of tarsomeres 2–4; tarsomeres 1–4 progressively shorter; distal tarsomere (5) distinctly shorter than combined length of tarsomeres 1–4 (Fig. 6A). Tarsomeres 1–4 each with a small euplantula, absent on tarsomere 5 (Fig. 6A). Pretarsus with well developed unguitractor plate; arolium large with broad outer band covered with oval or rounded outgrowths (Fig. 6B–C); pretarsal claws distinctly pectinate (Fig. 6D). Euplantulae without transverse furrows and lacking sensory bristles (Fig. 6E); surface microstructure smooth with irregular patterns of microgrooves (Fig. 6F).

**Figure 7.**
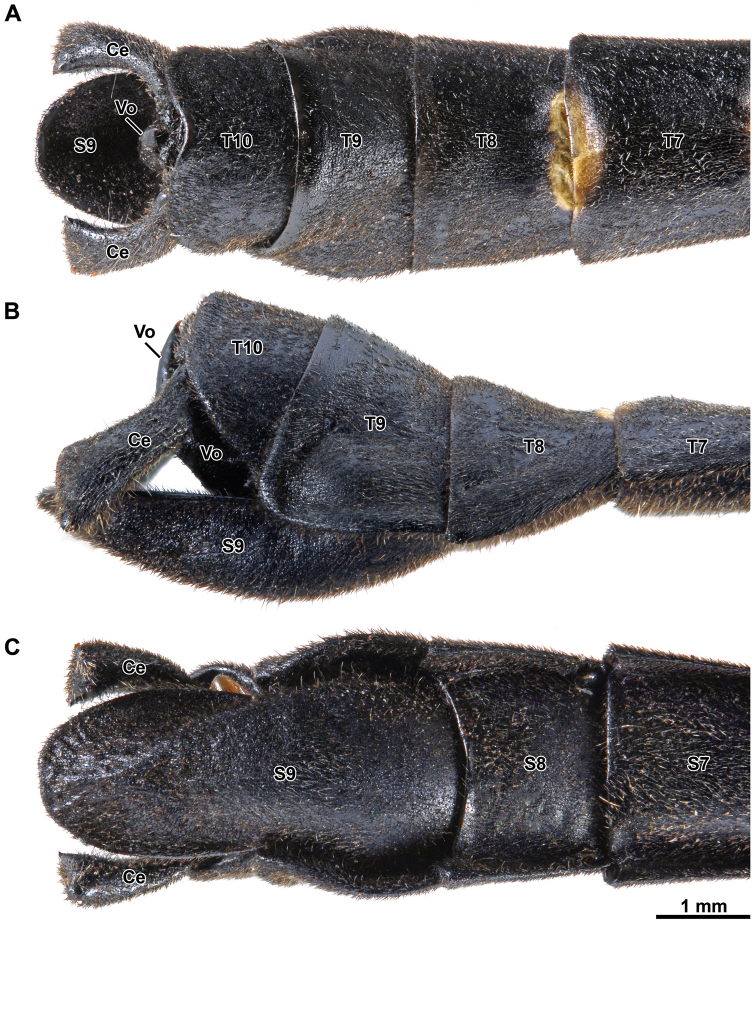
Orthomeria (Orthomeria) kangi sp. n. ♂ postabdomen **A** dorsal view **B** lateral view **C** ventral view; Ce, cercus; T7-T10, terga 7-19; S7-S9, sterna 7-9; Vo, vomer.

**Figure 8.**
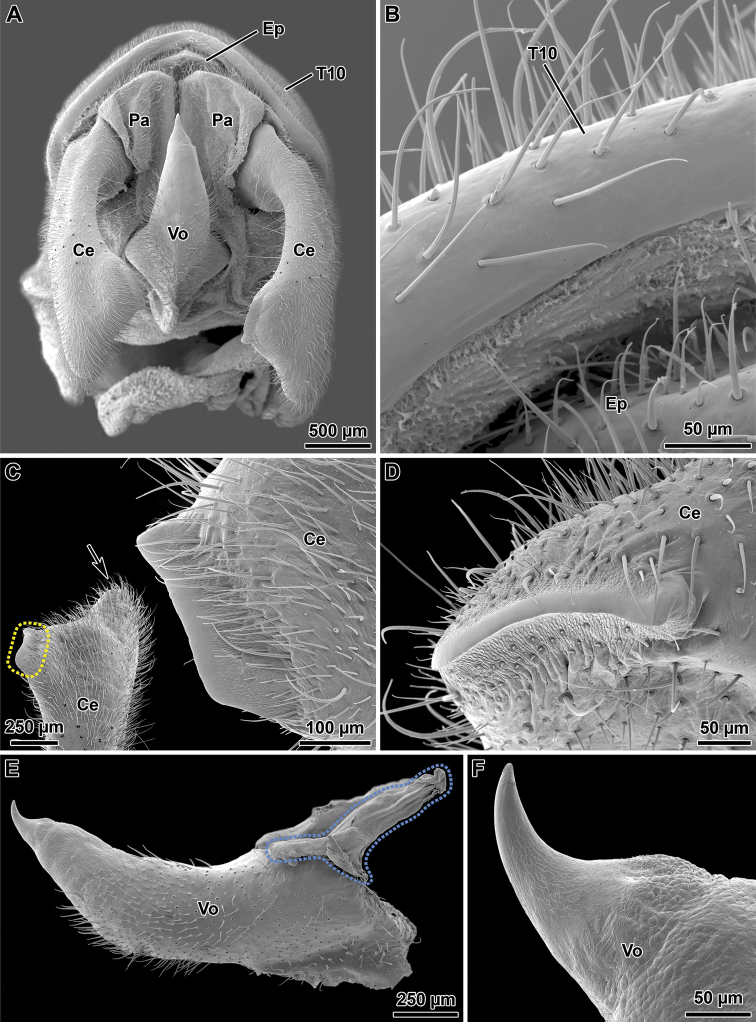
Orthomeria (Orthomeria) kangi sp. n. ♂ details of tenth abdominal segment **A** Whole segment X, ventral view **B** Hind margin of tergum 10, ventral view **C** Distal tip of right cercus with highlight (hatched area) and detail of the inner blade-like ridge, dorsal view, arrow indicates the outer pointed lobe **D** Blade-like ridge of right cercus, lateral view **E** Vomer, lateral view, hatched area indicates the proximal arm-like process **F** Spine-like apex of vomer, lateral view; Ce, cercus; Ep, epiproct; Pa, paraproct; T10, tergum 10; Vo, vomer.

Abdomen: About 1.60 times length of head and thorax combined. Segments 2–5 increasing in length, 6–9 decreasing in length, 10 about 1.40 times length of 9. First tergum (= median segment) longer than wide, and longer than metanotum, fused to the metanotum. Terga 2–6 longer than broad; terga 7–10 broader than long (Fig. 7A). Sternum 1 fused with the metasternum. Sterna without carinae. Sternum 9 undivided, upcurving and slightly boat shaped in lateral view (Fig. 7B), about 2.50 times length of sternum 8 (Fig. 7C), apex rounded distinctly projecting beyond segment 10. Tergum 10 slightly longer than tergum 9, hind margin concave dorso-medially (Fig. 7A). Tergal thorn pads lacking ventrally (Fig. 8A–B). Epiproct very short, triangular; paraprocts sub-triangular with a straight inner side (Fig. 8A). Cerci about 1.30 times length of tergum 10 (Fig. 7B), slightly flattened and gently incurved, clasper-like in appearance (Figs 7A–C, 8A). Distal tip of cerci broadened, vaguely bilobed; outer lobe roundly pointed, inner lobe differentiated into a sclerotized blade-like ridge ca. 340–360 µm in length (Fig. 8C–D). Vomer acutely triangular and setose (Figs 7A–B, 8A), inserted into sternum 10 through two proximal arm-like processes (Fig. 8E); proximal two-thirds almost straight, distal third strongly curved upwards with smooth spine-like apex (Figs 7A–B, 8E–F).

#### Description of the female.

**Figure 9.**
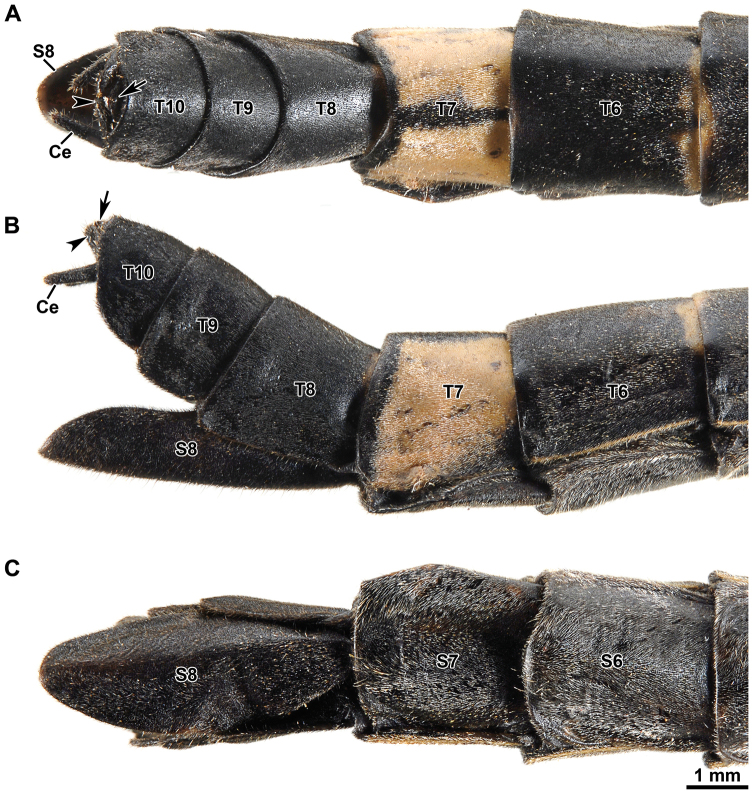
Orthomeria (Orthomeria) kangi sp. n. ♀ postabdomen **A** dorsal view **B** lateral view **C** ventral view; Ce, cercus; T6–T10, terga 6–10; S6–S8, sterna 6–8.

The female differs from the male in the following characters. Body slightly larger (Table 1) and more robust (Fig. 2B). Colouration variable, typically similar to male, except abdominal tergum 7 pale brown with a definite dark longitudinal line medially and lateral margins of abdominal terga with yellowish rim (Figs 2B, 9A–B). Alternatively, body, compound eyes, antennae and legs brown; costal region of hind wings dark brown with pale brown venation, anterior margin with a blood red tinge near wing articulation (Suppl. material 1). Mesothorax parallel-sided. Length of hind wing variable, reaching to posterior margin of the fourth tergum up to extending midway on to the fifth tergum. Abdomen about 1.50 times length of head and thorax combined. Abdominal segments 2–3 increasing in length, 4 shorther than 3, 5–6 increasing in length, 7–10 progressively shorter, 10 ca. 0.90 times length of 9. Abdominal terga 2–10 broader than long in full-grown females. Hind margin of tergum 10 rounded (Fig. 9A). Cerci terete and straight (Fig. 9A–B). Sternum 8 (= operculum) folded in two along the middle, covering completely the ovipositor, apex roundly pointed (Fig. 9B–C).

#### External eggshell morphology.

**Figure 10.**
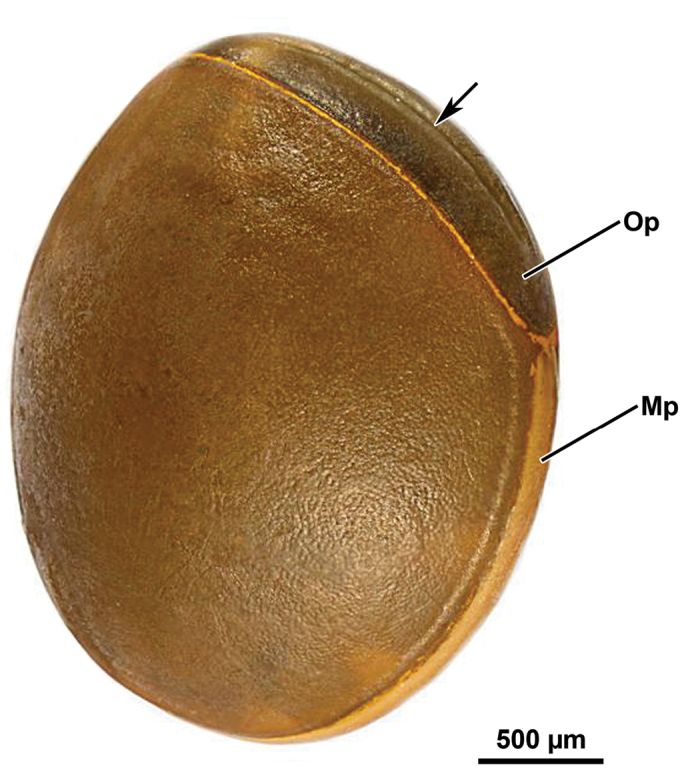
Orthomeria (Orthomeria) kangi sp. n. egg; Mp, micropylar plate; Op, operculum; Arrow, medial longitudinal furrow.

Capsule light brown, oval in outline, laterally flattened, surface minutely pitted (length, 2.6–2.7 mm; height, 2.3 mm; width, 1.6–1.7 mm) (Fig. 10). Operculum mid brown, elongate-oval with a medial longitudinal furrow, slightly convex in lateral aspect (height, 1.8 mm; width 0.7 mm). Opercular angle negative. Micropylar plate visible in lateral aspect, structured as pale brown stripe surrounding the entire capsule and delimited by a thin yellow rim extending also along the opercular opening area. Micropylar cup close to the posterior pole.

#### Description of the first-instar nymph.

**Figure 11.**
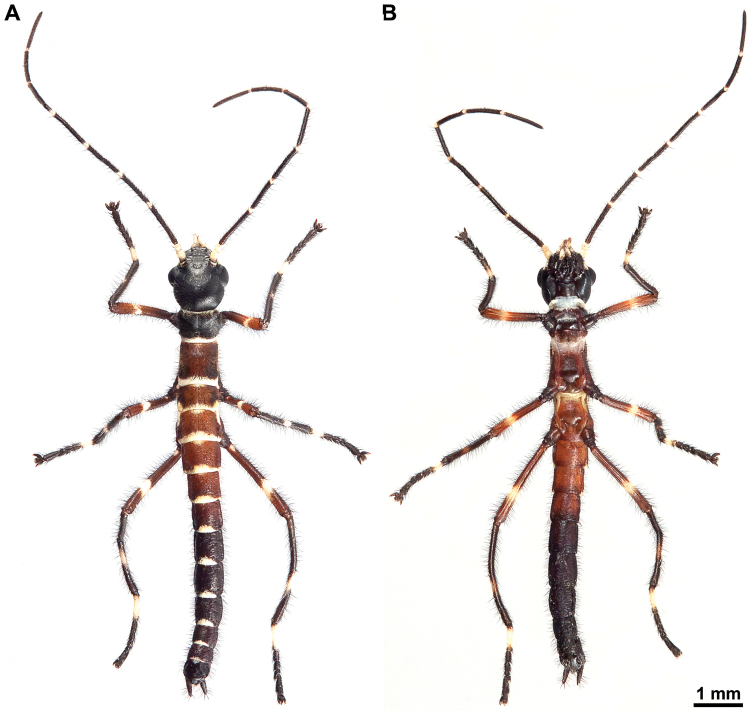
Orthomeria (Orthomeria) kangi sp. n. first instar nymph **A** Dorsal view **B** ventral view.

Body length ca. 8.9 mm. Head, prothorax, and abdominal segments V–X black; meso- and metatorax, and abdominal segments I–IV brown (Fig. 11A–B). Scape and pedicel white; flagellomeres black with white distal dot. Palpomeres of labial and maxillary palps white. Hind margin of thoracic and abdominal terga white. Femora brown with a white spot in the middle. Tibiae black with a central white band. Tarsi with proximal tarsomere white, remainder black.

#### Geographic distribution.

**Figure 12.**
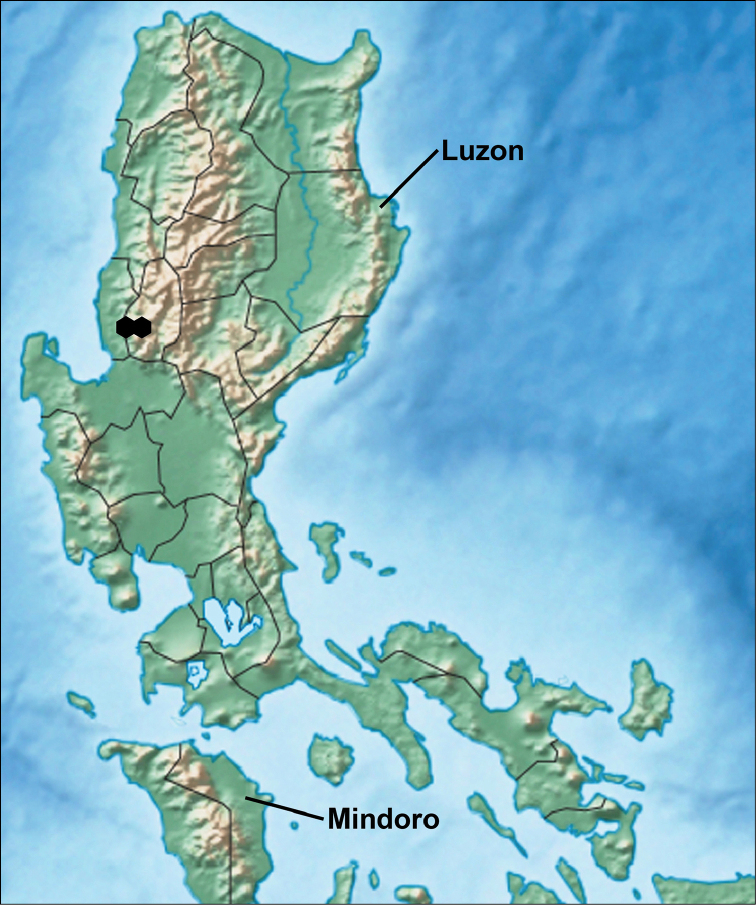
Distribution of Orthomeria (Orthomeria) kangi sp. n. in Luzon island.

The new species is so far reported only from the Benguet province, Luzon island, Northern Philippines (Fig. 12). Specimens have been found in the Sablang region (Barangay Bayabas, Mt. Bilbil) and in the Tuba region (Mt. Calugong).

#### Natural history observations.

**Figure 13.**
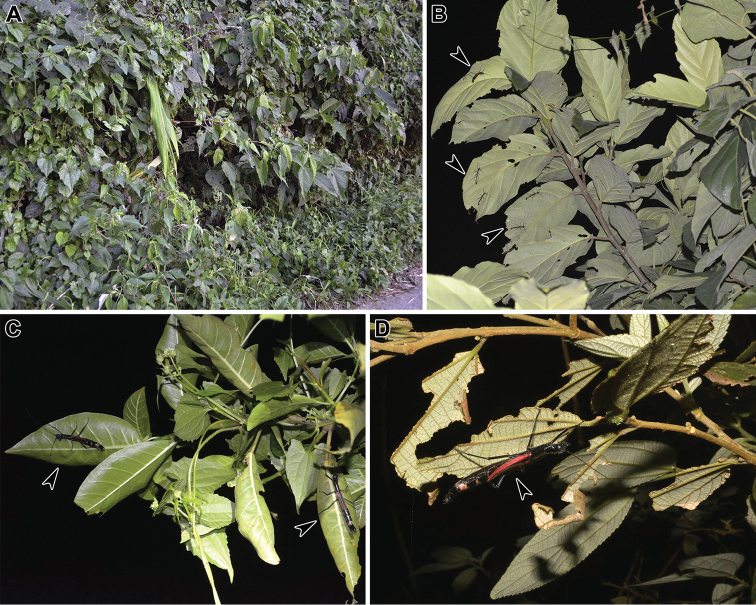
Orthomeria (Orthomeria) kangi sp. n. **A** Secondary vegetation in the type locality, Sablang, Benguet, Philippines **B** Adults on *Ficus* sp. **C** Adult females on *Ficus septica* **D** Adult female on *Pipturus arborescens*; arrowheads indicate the insects on branches. Photographs by Albert Kang.

The studied locality, Sablang, is a mountainous region (400-650 m elevation) of the Benguet Province, in the north-west Philippines. It is characterised by the presence of small communities scattered around a main provincial road, with several pockets of secondary vegetation (Fig. 13A) and some areas of primary forests. Adults of *Orthomeria kangi* sp. n. were first observed at night on the leaf underside of *Ficus* spp. trees (Moraceae). The host plants were mainly big trees of an unidentified *Ficus* sp. ca. 8–10 m height and up to 10–15 m wide located on forested slopes. The stick insects have been found on the low hanging branches (< 4 m height), where the number of observed individuals varied from 1 to 10 per tree (Fig. 13B). The species was found also in smaller fig trees (e.g. *Ficus septica*) ca. 2–3 m height (Fig. 13C), and occasionally on shrubs of *Pipturus* spp. and *Leucosyke* spp. (Urticaceae) (Fig. 13D), with usually 2–5 individuals present on the same plant. Daytime search revealed the presence of fewer individuals, mainly juveniles at different nymphal stages. Searching over a wide area, we noted that the distribution of the species on host trees was markedly discontinuous, with individuals concentrated on single larger plants and apparently absent from *Ficus* trees in the immediate vicinity.

Eggs were dropped to the ground and needed ca. 40 days to hatch at 23 °C. Newly hatched nymphs were reared to adulthood using the hauili tree (*Ficus septica*) or stinging nettle (*Urtica dioica*) as food plants. Under rearing conditions, the nymphal development lasted for ca. 60 days, and the average life span for males was ca. 45 days compared with ca. 140 days for females. The insects were active both during the day and night.

**Figure 14.**
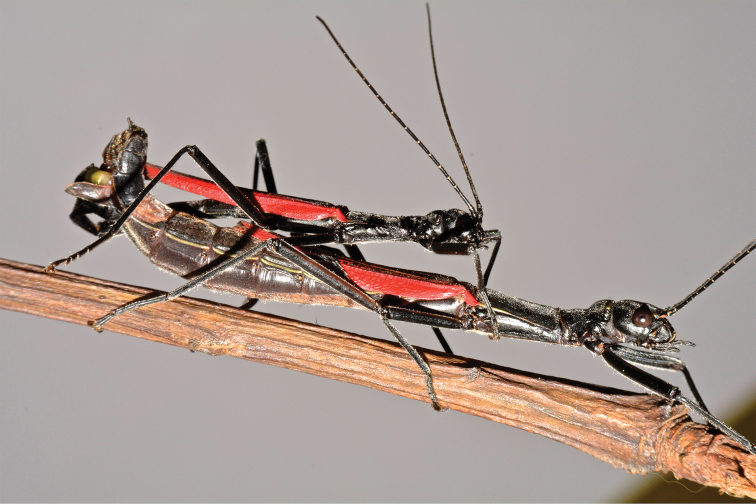
Orthomeria (Orthomeria) kangi sp. n. mating pair. Photograph by Albert Kang.

The mating was observed under rearing conditions. On day before the final moult, the subadult female starts to be guarded by 3–4 competing males, usually with one male mounted on the female’s back and facing into the same direction as the female. Copulation starts immediately after the female has completed the last nymphal moult. Generally, we found that when presented with a receptive virgin female, the male quickly mounts her and starts a series of abdominal bending movements apparently searching for the appropriate mating position. The female curve the abdominal tip upwards thereby exposing her terminalia, while the male bends its abdomen on the left side with his terminalia directed forward. The clasping cerci of the male grasp the female at the base of her eighth sternum, and at the same time a bulb-like phallic organ comes into contact with the female genitalia (Fig. 14). Copulation lasted approximately three hours. The presence of a spermatophore has not been ascertained. Mated females showed an aggressive behaviour towards potential mates, and multiple matings were rarely observed. They chase away approaching males through quivering movements of the body, beating legs on the substrate, and flashing their wings for a few seconds. This behaviour is also practiced by resting individuals when disturbed by conspecifics.

If threatened, adults and nymphs of *Orthomeria kangi* sp. n. spray a milky defensive secretion from the prothoracic exocrine glads and inevitably let themselves fall to the floor and quickly run away.

### Taxonomic notes on some species of Orthomeria (Orthomeria)

During our comparative analysis of the type material and additional specimens of *Orthomeria* spp., we recognised that *Orthomeria catadromus* (Westwood, 1859) represents a junior synonym of *Orthomeria pandora* (Westwood, 1859), syn. n. Both species were published in the same publication (Westwood 1859), but *Orthomeria pandora* has page priority. The type series of *Orthomeria catadromus* consists of three syntypes, two of them in BNHM and one in UMO. A specimen in BNHM bearing the following data is here designated as lectotype: the locality Sumatra was handwritten by Westwood on the identification label under *Aschiphasma catadromus*; the specimen has a white database label with the code BMNH(E) #845139. There is no separate label with a locality, however there is a small round grey label with a question mark. Although *Orthomeria catadromus* was originally recorded from Sumatra, there is reasonable doubt that this is in error, and subsequent reports of this species refer only to the Philippines (Bruner 1915, Bragg 2001). In fact, various species described by Westwood have doubtful locality data and several have since been recorded from different localities. Another example concerning the Philippine phasmid fauna is that of *Theramenes olivaceus* (Westwood, 1859), a species of Eubulidini (Heteropterygidae: Obriminae) originally described from Sri Lanka, but afterwards recorded from the Philippines (Bruner 1915) in accordance with the fairly restricted distribution of the tribe, not including Sri Lanka and continental Asia.

A lectotype is here designated also for *Orthomeria pandora* (Westwood, 1859). The original syntype series in BMNH originates from different localities. Two specimens originate from the Philippines and a third one from “ceram”. The specimens from the Philippines do not bear more precise data than “Philippine Islands”. The specimen from the Philippines still having one of its forelegs is hereby designated as lectotype.

### Identification key to the species of Orthomeria (Orthomeria)

♂♂

**Table:** 

1	Hind area of wings with at the base a large sky blue area	**2**
–	Hind area of wings uniformly dark or turning pale at the base without a distinct large sky blue area	**3**
2	Wings long, sky blue region not circular, beginning at A6; base of A 1–5 not blue	**Orthomeria (Orthomeria) superba (Redtenbacher, 1906)**
–	Wings short, base with an almost circular sky blue area which crosses all the anal veins	**Orthomeria (Orthomeria) versicolor (Redtenbacher, 1906)**
3	short winged species, wings projecting slightly over abdominal tergum 5	**4**
–	long winged species, wings projecting over abdominal tergum 7	**5**
4	Costal area of wings with definite orange area	**Orthomeria (Orthomeria) forstenii (Haan, 1842)**
–	Costal area of wings with a definite blood red area	**Orthomeria (Orthomeria) kangi sp. n.**
5	Mesonotum more than one and a half times as long as the pronotum	**Orthomeria (Orthomeria) pandora (Westwood, 1859)**
–	Mesonotum not one and a half times the length of pronotum	**6**
6	Anterior portion to radial vein of hind wing uniformly yellow. Base of antennae black, thereafter rust coloured	**Orthomeria (Orthomeria) smaragdinum (Redtenbacher, 1906)**
–	Anterior portion to radial vein of hind wing yellow with black cells. Antennae uniformly black	**Orthomeria (Orthomeria) xanti (Redtenbacher, 1906)**

♀♀*

**Table:** 

1	Hind area of wings with at the base a large sky blue area	**2**
–	Hind area of wings uniformly dark or turning pale at the base without a distinct large sky blue area	**3**
2	Wings long, sky blue region not circular, beginning at A6; base of A 1–5 not blue	**Orthomeria (Orthomeria) superba (Redtenbacher, 1906)**
–	Wings short, base with an almost circular sky blue area which crosses all the anal veins	**Orthomeria (Orthomeria) versicolor (Redtenbacher, 1906)**
3	Body > 50mm, tegmina brown, costal area of hind wings without red markings, tergum 7 pale without black longitudinal line	**Orthomeria (Orthomeria) pandora (Westwood, 1859)**
–	Body < 50mm , tegmina red, costal area of hind wings with red markings, tergum 7 pale with a definite black longitudinal line centrally	**Orthomeria (Orthomeria) kangi sp. n.**

* Females of Orthomeria (Orthomeria) forstenii, Orthomeria (Orthomeria) smaragdinum, and Orthomeria (Orthomeria) xanti are unknown.

## Discussion

### Phylogenetic interpretation of morphological characters

*Orthomeria kangi* sp. n. shows some phylogenetically informative characters that are helpful to find its placement among the subgroups of Euphasmatodea. The unbranched radial vein (= absence of the radial sector), the undivided sternum 9 and the incurved cerci with an apical spine or tooth in the male, represent key synapomorphies of Aschiphasmatidae (Bradler 2009). The latter specialized character is definitely homologous with the sharp blade-like ridge found in the male cerci of the new species. *Orthomeria kangi* sp. n. also shows distinctly pectinate claws, a putative derived character of the tribe Aschiphasmatini (Aschiphasmatidae excluding *Dajaca*) (Bragg 2001, Bradler 2009). Within Aschiphasmatidae, a brightly coloured (yellow, orange, or red) costal region of the hind wings occurs only in *Orthomeria*. This character can be interpreted as an autapomorphy that provides evidence for the monophyly of the genus.

Attachment structures of Aschiphasmatidae has been previously analysed in *Dallaiphasma eximius* (Gottardo 2011b). A feature shared between this species and *Orthomeria kangi* sp. n. is the absence of an euplantula on tarsomere V. In *Timema*, a small euplantula is present on the distal tarsomere (Beutel and Gorb 2008). Within Euphasmatodea the condition appears variable: the euplantula is present and generally small compared to the size of tarsomere V in *Eurycantha calcarata* and *Conlephasma enigma* (Gottardo and Heller 2012, Gottardo et al. 2015), while it is absent in several taxa including *Carausius morosus*, *Medauroidea extradentata*, *Ophicrania conlei*, and *Hermarchus leytensis* (Gottardo 2011a, Bußhardt et al. 2012, Gottardo and Vallotto 2014). The two species of Aschiphasmatidae have also smooth euplantulae on tarsomeres I-IV. Interestingly, while *Dallaiphasma eximius* has a honeycomb micropattern, *Orthomeria kangi* sp. n. shows only faint microgrooves on the contact surface. It has been hypothesized that nubby euplantulae covered with acanthae are a groundplan feature of Phasmatodea (Beutel and Gorb 2008), implying secondary modification of these surface structures in Aschiphasmatidae. It would be interesting to analyze the attachment structures of additional species of Aschiphasmatidae, since the character system could be more diverse within the family.

The male terminalia of *Orthomeria kangi* sp. n. show a number of specific modifications. An unusual feature is the complete absence of clasping devices (the tergal thorn pads) on the hind margin of the tergum X. Within Aschiphasmatidae these structures have been described in *Dallaiphasma eximius* as a single row of ca. 12 tooth-like projections (see Gottardo 2011b: fig. 5), and are usually present and well developed in Euphasmatodea (Bradler 2009). However, they are absent for example in *Timema*, *Agathemera*, in the taxon Sermyleformia sensu Bradler (2009), and their secondary loss has been established in extant leaf insects (partim) (Wedmann et al. 2007). It is conceivable that in *Orthomeria kangi* sp. n the reduction of tergal thorn pads has been compensated by the acquisition of the specialized cerci, that together with the vomer form the male clasping organs of this species. The structure of male terminalia has been described here in detail for the first time in *Orthomeria*, and for future studies these characters may represent important diagnostic features of males of species of this genus, as also exemplified in other euphasmatodean taxa by Bradler (2009) and Buckley et al. (2014).

### Intraspecific morphological variations

The captive rearing showed the presence of substantial intraspecific colour variation in the females of *Orthomeria kangi* sp. n. All wild individuals, both males and females, had the chromatic characters of the typical black colour morph. When the species was reared at cool temperatures (ca. 16 °C) all females developed the brown colour morph, while males were invariably black. Interestingly, the offspring of the brown females reared at warmer temperatures (ca. 23 °C) consisted of only black females.

A certain amount of variation was found also as to the length of the hind wings of females, regardless of the two colour morphs. In some females the hind wings reach the hind margin of the abdominal segment IV, whereas in other they can extend up to the hind margin of segment V. Intraspecific trends of variation in wing length have been rarely documented in Phasmatodea. A different example is that of *Asceles margaritatus* Redtenbacher, 1908 (Necrosciinae), where two separated macropterous and micropterous forms involving both sexes have been described (Bragg 2002).

### Foodplants of *Orthomeria*

Information about *Orthomeria* foodplants are mainly available for two species of the subgenus *Parothomeria*. Both Orthomeria (Parothomeria) alexis and Orthomeria (Parothomeria) cuprinus use trees of the genus *Macaranga* (Euphorbiaceae) as foodplants (Junker et al. 2008, Shimizu-kaya and Itioka 2015). For members of the subgenus *Orthomeria*, Bragg (2001) reported *Oreocnide rubescen* (Urticaceae) as foodplant of Orthomeria (Orthomeria) superba from Borneo. *Orthomeria kangi* sp. n also feeds on members of Urticaceae, but seems to prefer *Ficus* spp. (Moraceae) which represents a new foodplant association for *Orthomeria*.

### The species of *Orthomeria* from the Philippines

As a result of this study, seven species of Orthomeria (Orthomeria) are recognised, two of which occur in the Philippines: Orthomeria (Orthomeria) pandora and Orthomeria (Orthomeria) kangi sp. n. Both species seem to be restricted to Luzon island. Orthomeria (Orthomeria) pandora is found in the Sierra Madre mountain range in east Luzon, while Orthomeria (Orthomeria) kangi sp. n. occurs in the Benguet province in west Luzon. It is likely that other species of *Orthomeria* will be discovered in the other island of the Philippine archipelago.

## Supplementary Material

XML Treatment for Orthomeria (Orthomeria) kangi

## Supplementary materials

Supplementary material 1 Intraspecific colour variations Data type: multimedia Explanation note: Brown colour morph of a female of Orthomeria (Orthomeria) kangi sp. n. This dataset is made available under the Open Database License (http://opendatacommons.org/licenses/odbl/1.0/). The Open Database License (ODbL) is a license agreement intended to allow users to freely share, modify, and use this Dataset while maintaining this same freedom for others, provided that the original source and author(s) are credited.Davide Vallotto, Joachim Bresseel, Thierry Heitzmann, Marco Gottardo
