# Supplementary material for: A black-and-red stick insect from the Philippines – observations on the external anatomy and natural history of a new species of Orthomeria
Source: Zookeys. 2016 Feb 3;(559):35–57. doi: 10.3897/zookeys.559.6281 (PMC4768271; doi:10.3897/zookeys.559.6281)
Supplement: Supplementary material 1 — Intraspecific colour variations [file zookeys-559-035-s001.pdf]

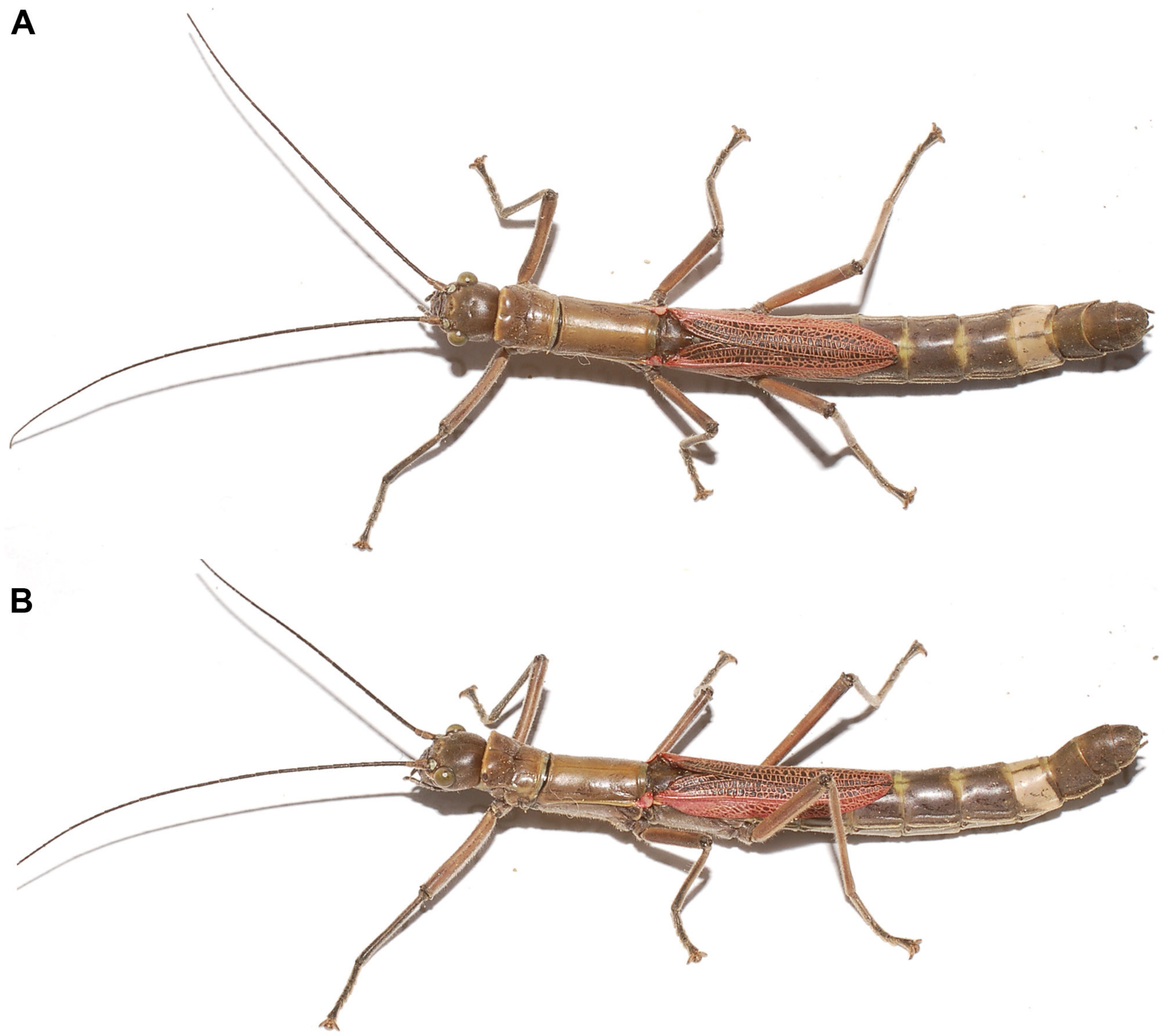


**Supplementary Figure S1.** *Orthomeria (Orthomeria) kangi* sp. n. ♀ paratype, brown colour morph **A** Dorsal view **B** Lateral view.
